# *Yupingfeng San* Inhibits NLRP3 Inflammasome to Attenuate the Inflammatory Response in Asthma Mice

**DOI:** 10.3389/fphar.2017.00944

**Published:** 2017-12-22

**Authors:** Xue Liu, Jiawen Shen, Danping Fan, Xuemei Qiu, Qingqing Guo, Kang Zheng, Hui Luo, Jun Shu, Cheng Lu, Ge Zhang, Aiping Lu, Chaoying Ma, Xiaojuan He

**Affiliations:** ^1^Institute of Basic Research in Clinical Medicine, China Academy of Chinese Medical Sciences, Beijing, China; ^2^School of Life Sciences and Engineering, Southwest Jiaotong University, Chengdu, China; ^3^Law Sau Fai Institute for Advancing Translational Medicine in Bone and Joint Diseases, School of Chinese Medicine, Hong Kong Baptist University, Kowloon Tong, Hong Kong; ^4^Institute of Clinical Medical Sciences, China-Japan Friendship Hospital, Beijing, China; ^5^School of Basic Medical Sciences, Shanghai University of Traditional Chinese Medicine, Shanghai, China

**Keywords:** *Yupingfeng San*, asthma, network pharmacology, NLRP3 inflammasome, inflammation

## Abstract

*Yupingfeng San* (YPFS) is a representative Traditional Chinese Medicine (TCM) formula with accepted therapeutic effect on Asthma. However, its action mechanism is still obscure. In this study, we used network pharmacology to explore potential mechanism of YPFS on asthma. Nucleotide-binding oligomerization domain (NOD)-like receptor pathway was shown to be the top one shared signaling pathway associated with both YPFS and asthma. In addition, NOD-like receptor family pyrin domain-containing 3 (NLRP3) inflammasome was treated as target protein in the process of YPFS regulating asthma. Further, experimental validation was done by using LPS-stimulated U937 cells and ovalbumin (OVA)-sensitized BALB/c mice model. *In vitro* experiments showed that YPFS significantly decreased the production of TNF-α and IL-6, as well as both mRNA and protein levels of IL-1β, NLRP3, Caspase-1 and ASC in LPS-stimulated U937 cells. *In vivo* experiment indicated that YPFS treatment not only attenuated the clinical symptoms, but also reduced inflammatory cell infiltration, mucus secretion and MUC5AC production in lung tissue of asthmatic mice. Moreover, YPFS treatment remarkably decreased the mRNA and protein levels of IL-1β, NLRP3, Caspase-1 and ASC in lung tissue of asthmatic mice. In conclusion, these results demonstrated that YPFS could inhibit NLRP3 inflammasome components to attenuate the inflammatory response in asthma.

## Introduction

Asthma is a chronic inflammatory disorder of the airways in which many inflammatory cells play a role. This disease is characterized by recurrent episodes of wheezing, breathlessness, chest tightness and coughing (Braman, 2006; Mims, 2015). The cause of asthma is unknown, but it is generally thought that gene-environment interactions have played important roles in risk factors (Ober and Yao, 2011; Bonnelykke and Ober, 2016). Previous study revealed that genetic predisposition and environmental exposures were most likely to cause inflammation in the lower airway (Bunyavanich and Schadt, 2015). Current research showed that Nucleotide-binding oligomerization domain (NOD)-like receptor family pyrin domain-containing 3 (NLRP3), one of nucleotide-binding domain and leucine-rich repeat containing (NLR) family membranes, had a substantial impact on tissue inflammation including respiratory diseases like asthma and could aggravate the progress of asthma (Kufer and Sansonetti, 2011; Im and Ammit, 2014; Hosseinian et al., 2015). For this reason, NLRP3 inflammasome has been suggested as an important target for inflammatory disease control (Peng et al., 2016). In addition, it has been reported that NLRP3 inflammasome activation could lead to the increased release of interleukin (IL)-1β (Haneklaus and O’Neill, 2015). There’s studies proved that NLRP3 mediated IL-1β could lead to a vicious cycle between previous and future exacerbations in asthma (Kim et al., 2017). For this reason, NLRP3 inflammasome and IL-1β have been suggested as important targets for inflammatory disease control (Fu et al., 2015; Peng et al., 2016).

Commonly, presently available drugs such as corticosteroids, biologic therapies have been found beneficial in controlling the asthma, but it had been proved with frequent side effects (Cooper et al., 2015). *Yupingfeng San* (YPFS), originally recorded in the book *Danxi Xinfa*, is a Chinese medicine formula consisting of *Astragalus membranaceus, Atractylodes macrocephala*, and *Saposhnikoviae radix*. YPFS has been found to have therapeutic effect on asthma and with less adverse reactions (Wang et al., 2013; Zhong et al., 2014; Chan et al., 2016). Modern pharmacological research indicated that YPFS could regulate the releases of cytokines, like IL-1β, necrosis factor alpha (TNF)-α and IL-6 (Du et al., 2013). However, the underlying mechanism of YPFS on asthma is still unknown and further inquiry is needed.

Traditional Chinese Medicine (TCM) is well-known for its multi-component, multi-target and multi-pathway. And most diseases are not simply caused by one single factor. The TCM treatments can be visualized as a complexity against complexity paradigm between multi-target therapy such as TCM and the complex biological networks of human diseases (Li et al., 2014). As a major method, network pharmacology provide a method to unravel the complex and holistic mechanisms of TCM in treating complex diseases (Goh et al., 2007). Moreover, successful attempts on TCM study have been achieved in our group by network pharmacology analysis (Niu et al., 2015; Zhao et al., 2015; Fan et al., 2016). Therefore, in this study, we explored the underlying mechanism of YPFS on asthma via network pharmacology analysis and experimental validation.

## Materials and Methods

### Network Pharmacology Analysis

Chemical compounds with OB ≥ 30% or DL ≥ 0.18 in *Astragalus membranaceus, Atractylodes macrocephala*, and *Saposhnikoviae radix* were obtained in Traditional Chinese Medicine Systems Pharmacology Database (TCMSP)^1^. Those compounds of the three drugs were combined and repeated compounds were deleted. The corresponding target of each component was acquired in TCMSP database. The corresponding gene name of each target protein was obtained in UniProt database^2^ and non-human target proteins were excluded. Finally, the target protein regulating by YPFS was acquired. Asthma-related human genes were searched on National Center for Biotechnology Information’s (NCBI) Gene database^3^. Then, the target genes of YPFS and asthma were mapped. The interaction protein of mapped gene was obtained through the String interaction database^4^ and the protein interaction network was constructed. The pathway enrichment of the interaction protein was used the Database for Annotation, Visualization and Integrated Discovery (DAVID)4^5^, and broad signaling pathways were eliminated. The top five pathways were extracted and the matching genes of the NOD-like receptor pathway which was ranked first were constructed the interaction network.

### Preparation of YPFS

All crude drugs were purchased from Beijing Tongrentang. 60 g *Astragalus membranaceus*, 20 g *Atractylodes macrocephala*, and 20 g *Saposhnikoviae radix* were extracted twice with boiling water, each time 40 min (1:6 and 1:4, w/v) (Song et al., 2013). Concentrating the two filtrates liquid into extract with electric thermostatic water bath and then transferred to the vacuum oven for drying. After drying, the solid extract was crushed into powder and stored at 4°C.

### Cell Culture

Human leukemic U937 cells were purchased from American Type Culture Collection (Manassa, VA, United States), and were cultured at 37°C in a humidified atmosphere with 5% CO_2_ in RPMI 1640 medium (GIBCO, Gaithersburg, MD, United States) supplemented with 10% fetal bovine serum (FBS) (GIBCO, Gaithersburg, MD, United States) and 1% penicillin-streptomycin (PS) (GIBCO, Gaithersburg, MD, United States).

### Cell Viability Assay

U937 cells were seeded at a density of 1.0 × 10^4^ cells/well into a 96-well with RPMI 1640 containing 5% FBS and 1% PS at 37°C in a 5% CO_2_ incubator. U937 were differentiated into macrophages by using phorbol 12-myristate 13-acetate (PMA) (Sigma, St. Louis, MO, United States) (0.01 μg/ml). After 48 h, the cells were washed with phosphate-buffered saline solution (PBS) twice and incubated with YPFS (0–100 μg/ml) for 45 h at 37°C in a 5% CO_2_ incubator. Cells were added with 10 μl CCK-8 reagent (Dojindo, Tokyo, Japan) into each well and further cultured for 3 h. The absorbance of each well was measured with a microplate reader (Bio-Tek Instruments, Winooski, VT, United States) at 450 nm.

### YPFS Treatment of U937 Cells

U937 cells were plated in 6 well plates (1 × 10^6^ cells/well) and incubated with PMA for 48 h at 37°C, 5% CO_2_. Then cells were washed for three times in PBS and induced with lipopolysaccharides (LPS) (Sigma, St. Louis, MO, United States) (0.1 μg/ml, 2 h). U937 cells were incubated with YPFS (25 μg/ml) for another 48 h. INF39 (Selleck, Shanghai, China), a non-toxic, irreversible NLRP3 inhibitor able to decrease interleukin-1β release from macrophages, was used as the positive control at a concentration of 10 μM (Cocco et al., 2017). The supernatant was collected to detect levels of IL-1β, TNF-α and IL-6 by ELISA and the cells were harvested to detect levels of NLRP3, ASC, Caspase-1 and IL-1β with Real-Time PCR and western blotting.

### Animals

Male BALB/c mice were purchased from Beijing Vital River Laboratory Animal Technology Co. Ltd., (Beijing, China), weighted 18–20 g with 7 weeks of age. The mice were bred in the China Academy of Chinese Medical Sciences [certification NO. SCXK (JING) 2016-0021] and housed in a room with a temperature-, humidity- and light-controlled environment. They were fed food and water ad libitum, and allowed to acclimatize themselves for 1 week before the initiation of experiment. The study was approved by the Research Ethics Committee of Institute of Basic Theory of Chinese Medicine, China Academy of Chinese Medical Sciences. All animals were treated in accordance with the guidelines and regulations for the use and care of animals of the Center for Laboratory Animal Care, China Academy of Chinese Medical Sciences.

### Induction of Asthma

To establish an asthma model, 0.5 ml mixture [10 μg ovalbumin (OVA), 2 mg sodium hydroxide and 0.5 ml saline] were injected on day 1, day 7, and day 14 for model group and administration group. The normal group was injected with the same amount of saline. From day 21, mice were sprayed with 1% OVA solution once a day for 30 min and sprayed for 12 days. The normal group was sprayed with saline.

### Drug Treatments

All the mice were divided into 4 groups: normal group, model group, dexamethasone (Dex) group (1 mg/kg/d) and YPFS group (13 g/kg/d), each group contained 10 mice. The dose of Dex and YPFS were reference to related literature (Dong et al., 2012; Wang et al., 2013), and the administration start from the atomization period (day 21). In the 30 min before the atomization, the mice of the positive drug group were injected with 0.2 ml of Dex. The mice in the administration group were given 0.5 ml of YPFS solution, and the mice in the model group and the normal group were given 0.5 ml of saline.

### Observation of Ethology

The ethology of the mice was evaluated by observation of the following parameters: Fur luster, touching of nose and scratching of ears, irritability, sneezing, rapid breathing and incontinence (Jia et al., 2017).

### Histological Analysis

Left lungs were removed from mice of each group, and distended with 10% buffered formalin. The tissues were sliced and embedded in paraffin, and 4 μm sections were prepared for morphological evaluation with hematoxylin-eosin staining (HE) and for mucopolysaccharide staining with alcian blue-periodic acid-schiff (AB-PAS). Sections from all of the left lobes were examined blindly by three individuals. The scoring system for cell infiltration was as follows: 0, no cells; 1, a few cells; 2, a ring of cells 1 cell layer deep; 3, a ring of cells 2–4 cells deep; and 4, a ring of cells > 4 cells deep. Goblet cell hyperplasia in the airway epithelium was quantified based on a five-point system: 0, no goblet cells; 1, <25% of the epithelium; 2, 25–50% of the epithelium; 3, 50–75% of the epithelium; and 4, >75% of the epithelium. Mean scores were calculated from ten mice (Underwood et al., 1995; Dong et al., 2012).

### Immunohistochemistry

Paraffin sections were deparaffinized in 100% xylene and rehydrated (100, 95, 90, 85, 80% ethanol). Endogenous peroxidase activity was blocked by treating with 3% hydrogen peroxide (10 min) followed by a PBS wash twice (5 min each). The sections were incubated with a MUC5AC antibody (Abcam, Cambridge, United Kingdom) at a 1:500 dilution in primary antibody dilution buffer (Beyotime, Shanghai, China) for 12 h at 4°C. The sections were washed three times (3 min each) in PBS, incubated with SignalStain Boost^®^ IHC Detection Reagent (HRP, Mouse) (Cell Signaling Technology, Beverly, MA, United States) for 30 min at room temperature, and washed in PBS for three times (5 min each). Secondary antibody was detected with SignalStain^®^ DAB Substrate Kit (Cell Signaling Technology, Beverly, MA, United States) Slides were counterstained with hematoxylin (Leagene, Beijing, China) and mounted. To evaluate the protein expression, semi-quantitative image analysis was employed to measure the mean optical density (MOD) using Image-Pro Plus 6.0 software.

### Bronchoalveolar Lavage Fluid (BALF) Cell Count

Bronchoalveolar lavage fluid was collected from the mice by endotracheal intubation using a catheter followed by lavage with 0.4 ml of PBS for three times as previously described (Mi Chun et al., 2017). After lavage, approximately 1 ml of BALF was recovered. Total BALF cells count was determined in a hemocytometer using trypan blue exclusion. Eosinophils, neutrophils, lymphocytes, and monocytes cell counts were determined in 300 BALF cells on cytospin smears of BAL samples from individual mice stained with Giemsa stain (Sigma, St. Louis, MO, United States).

### ELISA

Cell supernatants were collected as well as serum in mice were prepared. The levels of IL-1β, TNF-α and IL-6 were detected by using commercially available ELISA kits (eBioscience, San Diego, CA, United States) according to the manufacturer’s instructions.

### Real Time-PCR

The mRNA levels of IL-1β, NLRP3, cysteinyl aspartate specific proteinase (Caspase)-1 and advanced synthesis and catalysis (ASC) were analyzed by RT-PCR. Total RNA of lung tissue was isolated using TaKaRa MiniBEST Universal RNA Extraction Kit (TaKaRa, Kusatsu, Japan) according to the manufacturer’s instructions. This procedure was done under RNase-free conditions. The total RNA (1 μg) was reverse transcribed to cDNA using PrimeScript^TM^ RT reagent Kit with gDNA Eraser (TaKaRa, Kusatsu, Japan) according to the instruction manual. The specific transcripts were quantified by quantitative RT-PCR using SYBR^®^ Premix Ex Taq^TM^ II (TliRNaseH Plus), ROX plus (TaKaRa, Kusatsu, Japan) and analyzed with ABI 7500 RT-PCR system (Applied Biosystems, Foster, CA, United States). Gene-specific primers were synthesized by Sangon Biotech (Shanghai, China) and the following primer sequences were used: CATGAGTGCTGCTTCGACAT (forward) and GCTTCAGTCCCACACACAGA (reverse) for H-NLRP3, CTGACGGATGAGCAGTACCA (forward) and AGTCCTTGCAGGTCCAGTTC (reverse) for H-ASC, GCTTTCTGCTCTTCCACACC (forward) and TCCTCCACATCACAGGAACA (reverse) for H-caspase-1, CCATGGACAAGCTGAGGAAG (forward) and GTGATCGTACAGGTGCATCG (reverse) for H-IL-1β, TGGAGTCTACTGGCGTCTT (forward) and TGTCATATTTCTCGTGGTTCA (reverse) for H-GAPDH, GGAGGAAGAGGAGGAGGAAA (forward) and ACTGGAAGTGAGGTGGCTGT (reverse) for M-NLRP3, GGCTGCTGGATGCTCTGTA (forward) and AGGCTGGTGTGAAACTGAAGA (reverse) for M-ASC, CAGACAAGGGTGCTGAACAA (forward) and TCGGAATAACGGAGTCAATCA (reverse) for M-Caspase-1, TGGCAATGAGGATGACTTGT (forward) and TGGTGGTCGGAGATTCGTA (reverse) for M-IL-1β, AGGCCGGTGCTGAGTATGTC (forward) and TGCCTGCTTCACCACCTTCT (reverse) for M-GAPDH. The mRNA levels were normalized to GAPDH mRNA level. PCR conditions were shown as below: at 95°C for 30 s, 40 cycles at 95°C for 5 s and at 60°C for 30 s. Relative mRNA expression was calculated by comparative CT method.

### Western Blotting

The levels of NLRP3, ASC, Caspase-1 and IL-1β in mice were detected by western blotting. Tissue homogenates were prepared in lysis buffer (Beyotime, Shanghai, China), consisting of 1 nM phenylmethanesulfonyl fluoride (PMSF) (Beyotime, Shanghai, China). Proteins were denatured and equal amounts of proteins were electrophoresed in 12% bis-Tris/polyacrylamide gels (Beyotime, Shanghai, China) and transferred to Polyvinylidene fluoride (PVDF) (Amresco, Houston, TX, United States). Membranes with 0.45 μm pore-size Millipore filter. The membranes were blocked for 2 h in blocking solution (TBST containing 5% skim milk powder and 0.1% Tween 20) and incubated overnight at 4°C with anti-NLRP3, anti-Caspase-1, anti-IL-1β antibody (Abcam, Cambridge, United Kingdom) at 1:1000 and anti-ASC antibody (Abcam, Cambridge, United Kingdom) at 1:500 in TBST containing 5% bovine sera albumin. After that, incubation with horseradish peroxidase-conjugated secondary antibody (1:10000 dilution in TBST containing 5% skim milk powder) was performed at room temperature for 2 h, and immunoreactivity was detected by using enhanced chemiluminescence (APPLYGEN, Beijing, China). Blots were scanned and analyzed for measurement of the band intensities with UN-SCAN-IT version 5.1 software. Band intensity was calculated as follows: band intensity = sum of all pixel values in the segment selected-background pixel value in that segment.

### Statistical Analysis

Data were expressed as the mean ± SD of triplicate experiments and analyzed with SPSS version 17.0 software (SPSS Inc., Chicago, IL, United States). Statistically significant values were determined using ANOVA and Dunnett’s *post hoc* test, and *p*-values of less than 0.05 were considered statistically significant.

## Results

### YPFS Might Treat Asthma by Regulating the Expression of NLRP3 Inflammasome

**FIGURE 1:**
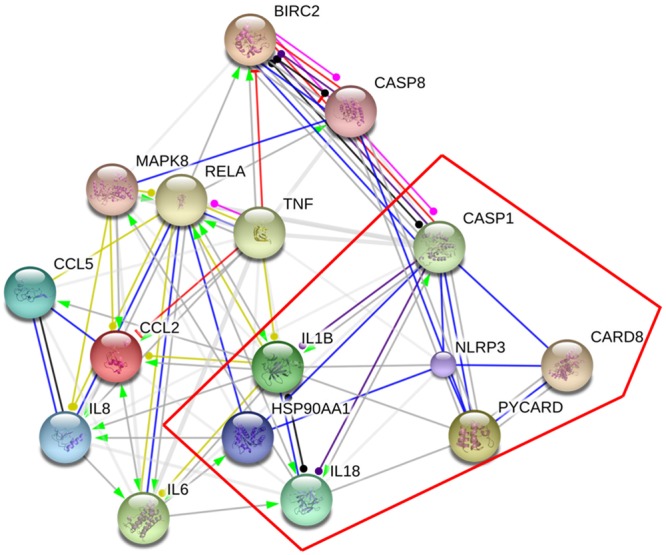
Target genes of YPFS treating asthma. The genes in the red box were the key genes of NLRP3 inflammasome.

Two hundred and thirty chemical compounds (Supplementary Tables 1–3), 372 human target proteins (Supplementary Table 4) and 793 asthma-related human genes (Supplementary Table 5) of YPFS were obtained in this study. The reaction network that YPFS antagonized asthma was built through String database (Supplementary Figure 1). DAVID database pathway enrichment analysis showed that the top five pathways of YPFS regulating asthma were NOD-like receptor signaling pathway, tumor necrosis factor (TNF) signal pathway, phosphatidylinositol-3-kinase (PI3K)-AKT signal pathway, Hypoxia-inducible factor (HIF)-1 signal pathway and nuclear factor-kappaB (NF-κB) signal pathway (**Table 1**). Using the String database to visualize the matching gene of the NOD-like receptor pathway, we found that the NLRP3 inflammasome might play an important role in the process that YPFS regulating the asthma (**Figure 1**).

**Table 1:** Top 5 shared signaling pathways of YPFS and asthma.

Pathway ID	Pathway description	False discovery rate	Observed gene count
4621	NOD-like receptor signaling pathway	1.69E-19	16
4668	TNF signaling pathway	2.00E-16	17
4151	PI3K-Akt signaling pathway	3.85E-15	24
4066	HIF-1 signaling pathway	9.63E-13	14
4064	NF-κB signaling pathway	2.32E-12	13

### YPFS Inhibited the Increase of Inflammatory Cytokines in LPS-Stimulated U937 Cells

**FIGURE 2:**
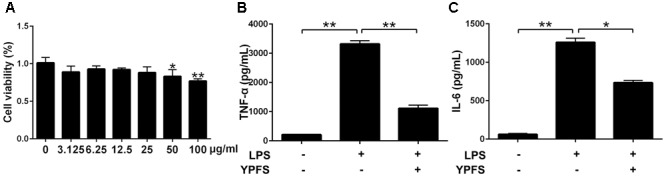
*Yupingfeng San* inhibited the increase of inflammatory cytokines in LPS-stimulated U937 cells. **(A)** Effect of YPFS on cell viability of U937 was detected by CCK-8 reagent. The OD values were measured at 450 nm. Cell supernatants were collected for ELISA detection. The levels of TNF-α **(B)** and IL-6 **(C)** were shown as above. *^∗^P <* 0.05, *^∗∗^P <* 0.01, compared with the model group.

Our results showed that YPFS didn’t exhibit obvious cytotoxicity at the concentration of 0, 3.125, 6.25 12.5 and 25 μg/ml (*P > 0.05*) (**Figure 2A**). Subsequently, 25 μg/ml YPFS was used for the further experiments. As shown in **Figures 2B,C**, We found that cytokine production in supernatant were all significantly increased in the LPS stimulation group compared to the control group (*P <* 0.01). YPFS treatment was able to interfere TNF-α and IL-6 production, which was shown by lower levels of TNF-α and IL-6 than the LPS stimulation group (*P <* 0.01).

### YPFS Reduced the Levels of NLRP3 Inflammasome Relative Genes and Proteins in LPS-Stimulated U937 Cells

**FIGURE 3:**
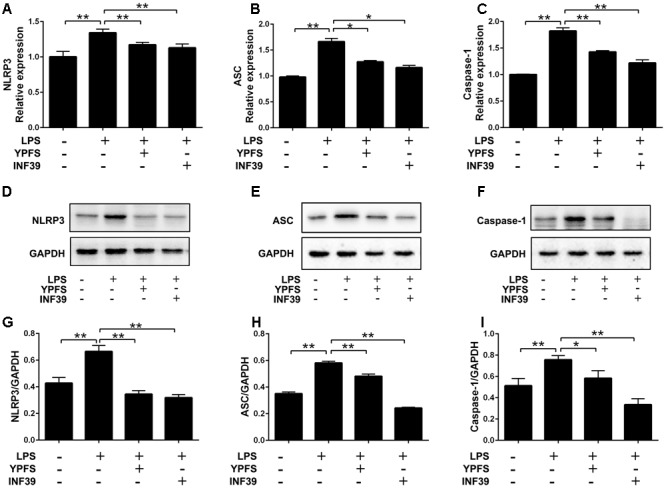
*Yupingfeng San* reduced the expression of NLRP3 Inflammasome in LPS-stimulated U937 cells. Cells were pre-treated with PMA for 48 h and then incubated with LPS for 2 h. Subsequently, cells were stimulated with YPFS. INF39 was used as the positive control. After 48 h, cells were collected. **(A–C)** The mRNA levels of NLRP3, ASC and Caspase-1 were detected by real time-PCR. **(D–I)** The protein levels of NLRP3, ASC and Caspase-1 were detected by western blotting, and the ratio of NLRP3/GAPDH, ASC/GAPDH and Caspase-1/GAPDH were shown. *^∗^P <* 0.05, *^∗∗^P <* 0.01, compared with model group.

To investigate whether YPFS regulate the expression of NLRP3 inflammasome, we detected the levels of NLRP3 inflammasome relative components. As shown in **Figure 3**, compared to the normal group, LPS stimulation significantly increased NLRP3, caspase-1 and ASC levels both at mRNA and protein levels (*P <* 0.01). Both YPFS and INF39 could significantly inhibit the mRNA and protein levels of NLRP3 inflammasome components compared to LPS stimulation group (*P <* 0.05 or *P <* 0.01).

### YPFS Inhibited the Production of IL-1β in LPS-Stimulated U937 Cells

**FIGURE 4:**
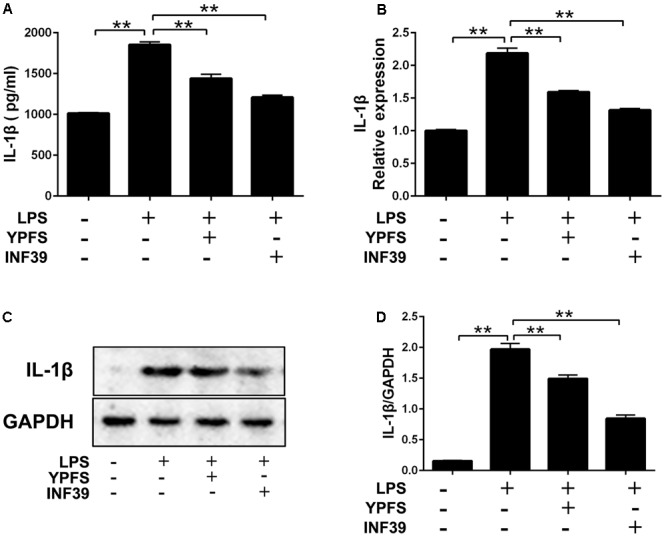
*Yupingfeng San* inhibited the production of IL-1β in LPS-stimulated U937 cells. **(A)** The level of IL-1β in cell supernatant was evaluated by ELISA. **(B)** The mRNA level of IL-1β in cells was assessed by real time-PCR. **(C,D)** The protein level of IL-1β in cells was detected by Western blotting. *^∗∗^P <* 0.01, compared with model group.

Compared to the normal group, LPS stimulation significantly increased IL-1β production (**Figure 4**). Both YPFS and INF39 could significantly inhibit the IL-1β production in the cell supernatants (**Figure 4A**). The mRNA (**Figure 4B**) and protein levels (**Figures 4C,D**) of IL-1β in LPS-stimulated U937 cells were also remarkably decreased (*P <* 0.01).

### YPFS Ameliorated Lung Inflammation, Mucus Secretion and MUC5AC Production in Asthma Mice

**FIGURE 5:**
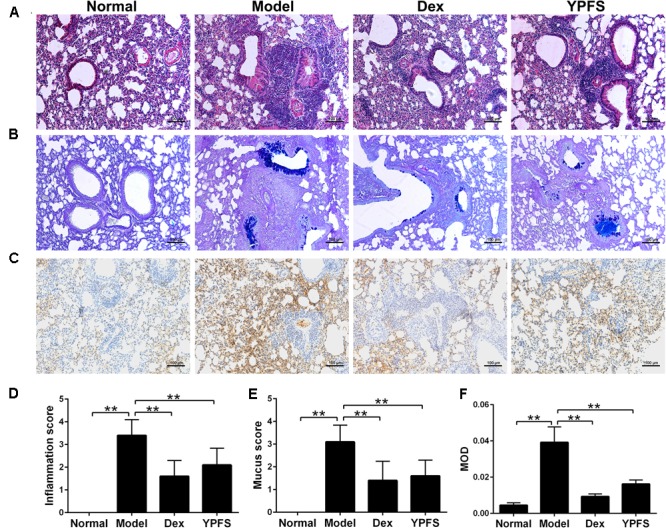
*Yupingfeng San* ameliorated lung inflammation, mucus secretion and reduced MUC5AC in asthma mice. Asthma model was induced by OVA-sensitized BALB/c mice. From the start of the atomization period, YPFS intragastric administration or dexamethasone (Dex) injection was carried out. **(A)** The upper lobe of left lungs was collected and the histopathological changes of the lung tissue were observed by HE staining. **(B)** The mucus secretion in the bronchus was observed by AB-PAS staining. **(C)** Immunohistochemical staining for MUC5AC. Tissue sections from lungs in each group were stained with monoclonal MUC5AC antibody. **(D–F)** The inflammation score, mucus secretion score and MOD in each group were shown. MOD: mean optical density. All the images are shown at 100× magnification. *^∗∗^P <* 0.01, compared with the model group.

In order to verify the therapeutic effect of YPFS on asthma, OVA-sensitized asthma model was constructed. After sensitization, mice in model group showed obvious symptoms of nose touching and ear scratching, irritability, sneezing, rapid breathing and incontinence, while these symptoms were all improved after using YPFS. As shown in **Figures 5A,D**, OVA induction caused the infiltration of inflammatory cells which were mainly eosinophils into the perivascular connective tissues and peribronchiolar compared with the normal group. YPFS significantly attenuated the eosinophil-rich infiltration compared with the model group. To further determine the effect of YPFS on mucus secretion, adjacent lung sections were stained with AB-PAS (**Figures 5B,E**). There was marked goblet cell hyperplasia and mucus hypersecretion within the bronchi in the mice lungs from the model group but not in the lungs from the normal group. Treatment with YPFS markedly attenuated the OVA-induced mucus secretion. MUC5AC is a major mucin protein secreted from the airway surface epithelium and played important role in asthma (Tan et al., 2011). To further confirm the effect of YPFS on asthma, histological sections of lung tissue were stained immunohistochemically to detect MUC5AC level. Our studies have proved that MUC5AC expression was markedly upregulated in lung tissue from OVA-induced asthmatic mice. Compared with normal group, MUC5AC were significantly increased in the stained sections of model groups. When compared with the model group, MUC5AC were significantly less in the stained sections of Dex group and YPFS group (**Figures 5C,F**).

### YPFS Decreased Inflammatory Cell Numbers in BALF

To determine the effect of YPFS on inflammatory cell infiltration in the lungs of asthmatic mice, the total numbers of inflammatory cells in the BALF of the YPFS-treated and untreated asthmatic mice were determined. Compared with the normal mice, mice in model group had significantly increased numbers of total inflammatory cells. By comparison, the YPFS- and Dex-treated mice had significantly fewer inflammatory cells in their BALF (**Table 2**). Analysis of the numbers of different inflammatory cells (eosinophils, neutrophils, macrophages, and lymphocytes) in the BALF showed that model group had very high numbers of eosinophils. Compared with model group, the YPFS-treated asthmatic mice had significantly fewer inflammatory cells, particularly eosinophils (**Table 2**).

**Table 2:** Effect of YPFS on bronchoalveolar lavage differential cell counts.

	Total cells (×10^3^)	Eosinophils (×10^3^)	Neutrophils (×10^3^)	Lymphocytes (×10^3^)	Macrophage (×10^3^)
Normal	50.71 ± 9.65	0.43 ± 0.17	0.53 ± 0.09	0.29 ± 0.06	48.46 ± 9.43
Model	371.54 ± 60.98^##^	117.91 ± 54.79^##^	5.48 ± 0.95^##^	5.97 ± 1.09^##^	241.32 ± 21.75^##^
Dex	200.31 ± 56.91^∗∗^	59.36 ± 27.78^∗^	2.56 ± 1.08^∗∗^	2.97 ± 1.51^∗∗^	110.27 ± 49.31^∗∗^
YPFS	263.66 ± 63.93^∗∗^	71.89 ± 26.51^∗^	3.29 ± 1.85^∗∗^	3.96 ± 1.46^∗∗^	183.51 ± 38.67^∗∗^

*^##^P < 0.01, compared with the normal group.*^∗^P <* 0.05, *^∗∗^P <* 0.01, compared with the model group.*

### YPFS Reduced Pro-inflammatory Cytokines Expression in Serum of Asthma Mice

**FIGURE 6:**
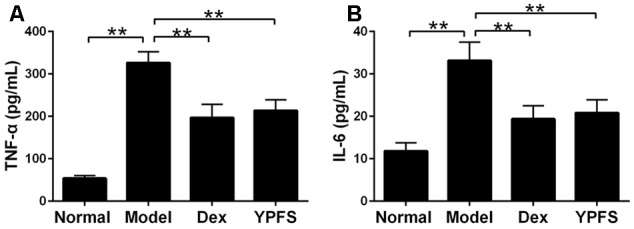
*Yupingfeng San* reduced pro-inflammatory cytokines expression in serum of asthma mice. Mice were sacrificed and the serum was collected for ELISA detection. The levels of TNF-α **(A)** and IL-6 **(B)** were shown as above. *^∗∗^P <* 0.01, compared with the model group.

To observe the effects of YPFS on production of pro-inflammatory cytokines in OVA-sensitized asthma mice, the expression of TNF-α and IL-6 in serum of OVA-sensitized asthma mice were examined by ELISA. Our results showed that levels of TNF-α and IL-6 in model group were all elevated. However, these effects were attenuated by YPFS treatment (**Figure 6**).

### YPFS Inhibited the Expression of NLRP3 Inflammasome in OVA-Sensitized Asthma Mice

**FIGURE 7:**
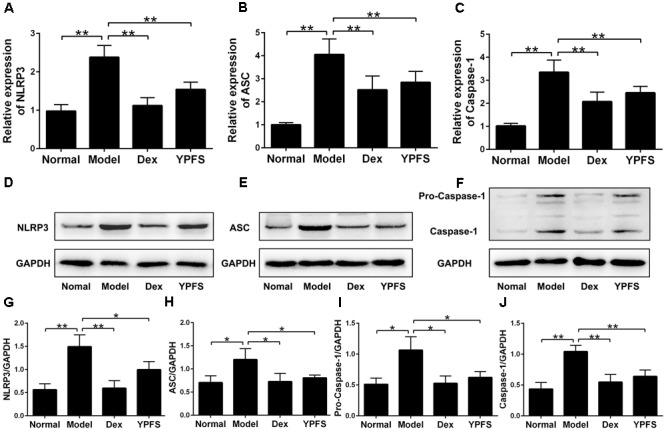
*Yupingfeng San* inhibited the expression of NLRP3 inflammasome in OVA-sensitized asthma mice. After mice were sacrificed, the upper lobe of the right lung was collected for RT-PCR and western blotting detection. **(A–C)** The mRNA levels of NLRP3, ASC and Caspase-1 on the upper lobe of the right lung were detected by RT-PCR. **(D–J)** The protein levels of NLRP3, ASC, Caspase-1 and Pro-Caspase-1 were detected by western blotting, and the ratio of NLRP3/GAPDH, ASC/GAPDH, Caspase-1/GAPDH and Pro-Caspase-1/GAPDH were shown. *^∗^P <* 0.05, *^∗∗^P <* 0.01, compared with the model group.

To further examine the mechanism of YPFS on OVA-sensitized asthma model, NLRP3 inflammasome level in lung tissues of mice in each group was detected. The results of RT-PCR showed that the mRNA levels of NLRP3, ASC and Caspase-1 in lung tissue was dramatically decreased after YPFS treatment compared to the model group (**Figures 7A–C**). Meanwhile, the protein levels of NLRP3, ASC, Caspase-1 and Pro-Caspase-1 in lung tissue of YPFS treated OVA-sensitized asthma mice also decreased significantly in comparison with those in model group (**Figures 7D–J**).

### YPFS Inhibited the Expression of IL-1β in OVA-Sensitized Asthma Mice

**FIGURE 8:**
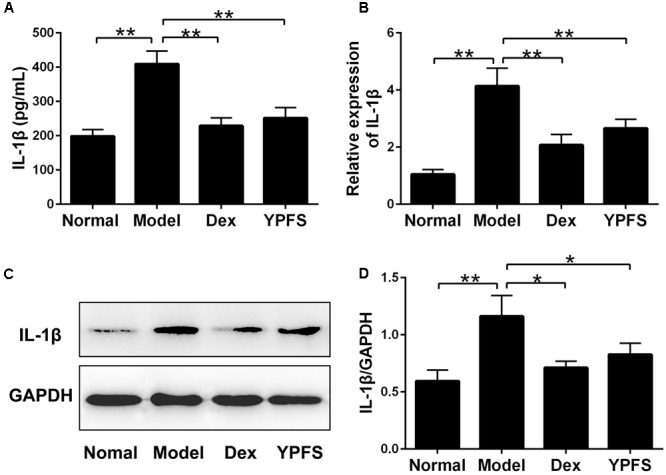
*Yupingfeng San* inhibited the expression of IL-1β in OVA-sensitized asthma mice. **(A)** The level of IL-1β in the serum was detected by ELISA. **(B)** The mRNA level of IL-1β on the upper lobe of the right lung was detected by RT-PCR. **(C,D)** The protein level of IL-1β was detected by western blotting and the ratio of IL-1β/GAPDH was shown. *^∗^P <* 0.05, *^∗∗^P <* 0.01, compared with the model group.

NLRP3 inflammasome activation could cause excessive IL-1β activation (Menu and Vince, 2011). To further verify the effects of YPFS on production of IL-1β, the level of IL-1β in serum was detected. As shown in **Figure 8A**, the level of IL-1β in the serum of model group was significantly higher than those in normal group. In contrast, higher level of IL-1β was significantly inhibited by YPFS treatment. As the level of IL-1β in serum was suppressed by YPFS, we wondered whether it could also be inhibited in the injured lung. As shown in **Figures 8B–D**, the mRNA and protein levels of IL-1β in the lung from model group were apparently raised compared to normal group, which was significantly decreased by YPFS treatment.

## Discussion

*Yupingfeng San* is widely used to treat the diseases of respiratory systems and immune systems, especially asthma (Li et al., 2013; Wang et al., 2013; Chan et al., 2016). In a previous clinical trial, patients’ cardinal symptoms of asthma were found reduced markedly after treated with YPFS (Chen et al., 2014). Previous studies also found YPFS had anti-inflammatory effect and this effect was achieved through suppressing the transcript and protein expressions of pro-inflammatory cytokine (Du et al., 2013; Sun et al., 2017). Consistent with previous researches, our study confirmed the therapeutic effect of YPFS on asthma. It could improve the symptoms of asthma mice like, nose touching, ear scratching, irritability, sneezing, rapid breathing and incontinence. After treating with YPFS, inflammatory cell infiltration, mucus secretion and MUC5AC expression in lungs of asthmatic mice were significantly inhibited. It also decreased inflammatory cell numbers in BALF and TNF-α, IL-6 levels in asthmatic mice plasma.

Although lots of researches had confirmed the efficacy of YPFS, more mechanisms by which YPFS inhibit inflammatory response should be fully elucidated (Chen et al., 2014; Song et al., 2016). In this study, we used network pharmacological analysis to investigate the mechanisms of YPFS acting on asthma. Our analysis indicated that multiple pathways were closely associated with asthma, such as TNF signaling pathway, PI3K-Akt signaling pathway, HIF-1 signaling pathway, and NF-κB signaling pathway. And these findings were consistent with previous studies (Crotty Alexander et al., 2013; Li et al., 2015, 2016; Schuliga, 2015; Peng et al., 2016). In addition, we found that NOD-like receptor signaling pathway was the top one shared signaling pathway of YPFS and asthma, which was not explored before. Studies on NOD-like receptor signaling pathways revealed that NLRP3, composed by NLRP3, ASC and Caspase-1, could be a very important immune receptor responsible for auto-inflammatory response (Martinon et al., 2009), and when excessive activated, NLRP3 inflammasome could lead to serious inflammatory conditions (Latz et al., 2013). Moreover, previous clinical and experimental studies demonstrated that the expression of NLRP3 inflammasome was up-regulated in patients with asthma and OVA-induced asthma model (Kim et al., 2017; Rossios et al., 2017), which indicated NLRP3 inflammasome played an important role in the pathology of asthma (Simpson et al., 2014). Our results showed that mRNA and protein levels of NLRP3, ASC and Caspase-1 in LPS-stimulated U937 cells as well as lung tissues of OVA-induced asthma mice were dramatically decreased after YPFS treatment, which suggested YPFS could inhibit the inflammatory response in OVA-induced asthma through suppressing the NLRP3 inflammasome.

In addition, more evidences implicated NLRP3 inflammasome activation could lead to the increased release of IL-1β (Negash et al., 2013; Kim et al., 2017). And this conclusion was also confirmed in patients with asthma (Simpson et al., 2014). Further research revealed that NLRP3 inflammasome-mediated IL-1β might be one of the key factors which could induce inflammation and result in asthma, and increasing IL-1β could also be seen as a marker of asthma (Kim et al., 2015; Giuffrida and Valero, 2017). Therefore, targeting elevated IL-1β instead of using corticosteroids might be an effective treatment for asthma. In this study, the expression of IL-1β in LPS-stimulated U937 Cells, in serum and in the lung tissue of asthma mice was all detected. We found that YPFS not only significantly inhibited the IL-1β production *in vitro*, but also decreased its levels in the periphery and lung tissue of OVA-induced asthma mice. These data indicated YPFS could inhibit inflammatory response in asthma through modulating NLRP3 inflammasome and subsequently down-regulating IL-1β.

In this study, we provided an integrative analysis by combining network pharmacology prediction with experiment validation to understand the pharmacological mechanism of YPFS acting on asthma. Furthermore, our finding also demonstrated that network pharmacology was a fairly reliable way to find TCM’s target and possible mechanisms.

## Conclusion

Our data in this research demonstrated that YPFS could attenuate inflammatory response in asthma through suppressing the NLRP3 inflammasome.

## Ethics Statement

Full name of the ethics committee that approved the study. The study was approved by the Research Ethics Committee of Institute of Basic Theory of Chinese Medicine, China Academy of Chinese Medical Sciences. Consent procedure used for human participants or for animal owners. Male BALB/c mice were purchased from Beijing Vital River Laboratory Animal Technology Co. Ltd., weighted 18–20 g with 7 weeks of age. The mice were bred and housed in a room with a temperature-, humidity- and light-controlled environment. They were fed food and water *ad libitum*, and allowed to acclimatize themselves for 1 week before the initiation of experiment. Any additional considerations of the study in cases where vulnerable populations were involved, for example minors, persons with disabilities or endangered animal species.

## Author Contributions

XL and JS performed the major research in equal contribution. DF, XQ, QG, KZ, HL, JS, CL, GZ, and AL provided the technical support. CM contributed to final approval of the version to be published. XH designed the study and revised the manuscript.

## Conflict of Interest Statement

The authors declare that the research was conducted in the absence of any commercial or financial relationships that could be construed as a potential conflict of interest. The reviewer YZ and handling editor declared their shared affiliation.

## Footnotes

1 http://ibts.hkbu.edu.hk/LSP/tcmsp.php

2 http://www.uniprot.org/

3 https://www.ncbi.nlm.nih.gov/genbank/

4 https://string-db.org/

5 https://david.ncifcrf.gov/
