# Supplementary material for: Yupingfeng San Inhibits NLRP3 Inflammasome to Attenuate the Inflammatory Response in Asthma Mice
Source: Front Pharmacol. 2017 Dec 22;8:944. doi: 10.3389/fphar.2017.00944 (PMC5743824; doi:10.3389/fphar.2017.00944)
Supplement: Supplementary file 1 [file Presentation_1.PDF]

## *Supplementary Material*

# *Yupingfeng San* inhibits NLRP3 inflammasome to attenuate the inflammatory response in asthma mice

Xue Liu[1,2†], Jiawen Shen[1,2†], Danping Fan[1], Xuemei Qiu[1,2], Qingqing Guo[1,3], Kang Zheng[1,3], Hui Luo[1,2], Jun Shu[4], Cheng Lu[1], Ge Zhang[3], Aiping Lu[3,5], Chaoying Ma[2*], Xiaojuan He[1,3*]

*Correspondence: Chaoying Ma, mcy195888@126.com; Xiaojuan He, hxj19@126.com

† These authors contributed equally to this work.

# 1 Supplementary Tables and Figures

## 1.1 Supplementary Tables

**Supplementary Table 1. Chemical compounds of *Astragalus Membranaceus***

| Mol ID | Chemical Compound | OB (%) | DL |
|---|---|---|---|
| MOL000114 | vanillic acid | 35.47 | 0.04 |
| MOL000131 | EIC | 41.9 | 0.14 |
| MOL000211 | Mairin | 55.38 | 0.78 |
| MOL000239 | Jaranol | 50.83 | 0.29 |
| MOL000296 | hederagenin | 36.91 | 0.75 |
| MOL000033 | (3S,8S,9S,10R,13R,14S,17R)-10,13-dimethyl-17-[(2R,5S)-5-propan-2-yloctan-2-yl]-2,3,4,7,8,9,11,12,14,15,16,17-dodecahydro-1H-cyclopenta[a]phenanthren-3-ol | 36.23 | 0.78 |
| MOL000354 | isorhamnetin | 49.6 | 0.31 |
| MOL000371 | 3,9-di-O-methylnissolin | 53.74 | 0.48 |
| MOL000372 | 3-Hydroxy-2-picoline | 62.47 | 0.02 |
| MOL000374 | 5'-hydroxyiso-muronulatol-2',5'-di-O-glucoside | 41.72 | 0.69 |
| MOL000378 | 7-O-methylisomucronulatol | 74.69 | 0.3 |
| MOL000379 | 9,10-dimethoxypterocarpan-3-O-β-D-glucoside | 36.74 | 0.92 |
| MOL000380 | (6aR,11aR)-9,10-dimethoxy-6a,11a-dihydro-6H-benzofurano[3,2-c]chromen-3-ol | 64.26 | 0.42 |
| MOL000381 | 13-hydroxy-9,11-octadecadienoic acid | 35.6 | 0.17 |
| MOL005928 | isoferulic acid | 50.83 | 0.06 |
| MOL000386 | Fucopyranose, L- | 42.51 | 0.03 |
| MOL000387 | Bifendate | 31.1 | 0.67 |
| MOL000389 | FERULIC ACID (CIS) | 54.97 | 0.06 |
| MOL000392 | formononetin | 69.67 | 0.21 |
| MOL000395 | GGB | 54.95 | 0.03 |
| MOL000397 | cis-p-Coumarate | 45.98 | 0.04 |
| MOL000398 | isoflavanone | 109.99 | 0.3 |
| MOL000401 | astragalosideI | 46.79 | 0.11 |
| MOL000403 | astragalosideII | 46.06 | 0.13 |
| MOL000405 | astragalosideIII | 31.83 | 0.1 |
| MOL000414 | Caffeate | 54.97 | 0.05 |
| MOL000417 | Calycosin | 47.75 | 0.24 |
| MOL000420 | XLS | 51.08 | 0.02 |
| MOL000421 | nicotinic acid | 47.65 | 0.02 |
| MOL000422 | kaempferol | 41.88 | 0.24 |
| MOL000424 | RAM | 50.5 | 0.04 |

| | | | |
|---|---|---|---|
| MOL000429 | Crystal VI | 83.96 | 0.02 |
| MOL000430 | betaine | 40.92 | 0.01 |
| MOL000432 | linolenic acid | 45.01 | 0.15 |
| MOL000433 | FA | 68.96 | 0.71 |
| MOL000434 | acetylastragaloside I | 43.54 | 0.09 |
| MOL000435 | acetylastragaloside I_qt | 30.75 | 0.17 |
| MOL000436 | (Z)-1-(2,4-dihydroxyphenyl)-3-(4-hydroxyphenyl)prop-2-en-1-one | 87.51 | 0.15 |
| MOL000438 | (3R)-3-(2-hydroxy-3,4-dimethoxyphenyl)chroman-7-ol | 67.67 | 0.26 |
| MOL000439 | isomucronulatol-7,2'-di-O-glucosiole | 49.28 | 0.62 |
| MOL000442 | 1,7-Dihydroxy-3,9-dimethoxy pterocarpene | 39.05 | 0.48 |
| MOL000054 | L- | 47.64 | 0.03 |
| MOL000061 | Prolinum | 77.57 | 0.01 |
| MOL000098 | quercetin | 46.43 | 0.28 |
| MOL000437 | Hirsutrin | 1.86 | 0.77 |
| MOL000423 | rhamnocitrin-3-O-glucoside | 2.87 | 0.76 |
| MOL000415 | rutin | 3.2 | 0.68 |
| MOL000396 | (+)-Syringaresinol | 3.29 | 0.72 |
| MOL000375 | 5'-hydroxyiso-muronulatol-2',5'-di-O-glucoside_qt | 3.65 | 0.8 |
| MOL000412 | Mucronulatol | 4.22 | 0.26 |
| MOL000406 | astragaloside III _qt | 5.35 | 0.32 |
| MOL000373 | (2S)-4-methoxy-7-methyl-2-[1-methyl-1-[(2S,3R,4S,5S,6R)-3,4,5-trihydroxy-6-methylol-tetrahydropyran-2-yl]oxy-ethyl]-2,3-dihydrofuro[3,2-g]chromen-5-one | 5.38 | 0.81 |
| MOL000377 | 7-hydroxy-3-(2-hydroxy-3,4-dimethoxy-phenyl)chromone | 5.45 | 0.3 |
| MOL000416 | Lariciresinol | 5.53 | 0.38 |
| MOL000410 | AstragalosideIV_qt | 7.07 | 0.32 |
| MOL000408 | astragalosideIV_qt | 7.07 | 0.32 |
| MOL000418 | 3'-Hydroxy-4'-methoxyisoflavone-7-O-beta-D-glucoside | 10.05 | 0.81 |
| MOL000391 | Ononin | 11.52 | 0.78 |
| MOL000404 | astragalosideII_qt | 11.55 | 0.25 |
| MOL000441 | LUPENONE | 11.66 | 0.78 |
| MOL001955 | Heriguard | 11.93 | 0.33 |
| MOL000356 | lupeol | 12.12 | 0.78 |
| MOL000402 | astragalosideI_qt | 12.34 | 0.2 |
| MOL000251 | Rhamnocitrin | 12.9 | 0.27 |
| MOL000399 | Docosanoate | 15.69 | 0.26 |
| MOL000376 | 7,2'-dihydroxy-3',4'-dimethoxyisoflavone-7-O-β-D-glucoside | 16.16 | 0.86 |
| MOL000400 | Flavaxin | 18.18 | 0.5 |

| | | | |
|---|---|---|---|
| MOL000411 | Astraisoflavanin | 18.37 | 0.86 |
| MOL000390 | daidzein | 19.44 | 0.19 |
| MOL000295 | alexandrin | 20.63 | 0.63 |
| MOL000440 | isomucronulatol-7,2'-di-O-glucosiole_qt | 23.42 | 0.79 |

**Supplementary Table 2. Chemical compounds of *Atractylodes Macrocephala***

| Mol ID | Chemical Compound | OB (%) | DL |
|---|---|---|---|
| MOL000018 | (+/-)-Isoborneol | 86.98 | 0.05 |
| MOL000019 | D-Camphene | 34.98 | 0.04 |
| MOL000020 | 12-senecioyl-2E,8E,10E-atractylentriol | 62.4 | 0.22 |
| MOL000021 | 14-acetyl-12-senecioyl-2E,8E,10E-atractylentriol | 60.31 | 0.31 |
| MOL000022 | 14-acetyl-12-senecioyl-2E,8Z,10E-atractylentriol | 63.37 | 0.3 |
| MOL000023 | Hemo-sol | 39.84 | 0.02 |
| MOL000025 | α-Longipinene | 53.26 | 0.12 |
| MOL000028 | α-Amyrin | 39.51 | 0.76 |
| MOL000030 | (1R)-2-methyl-1-phenylprop-2-en-1-ol | 75.1 | 0.03 |
| MOL000033 | (3S,8S,9S,10R,13R,14S,17R)-10,13-dimethyl-17-[(2R,5S)-5-propan-2-yloctan-2-yl]-2,3,4,7,8,9,11,12,14,15,16,17-dodecahydro-1H-cyclopenta[a]phenanthren-3-ol | 36.23 | 0.78 |
| MOL000038 | Akridin | 33.71 | 0.1 |
| MOL000039 | (1S,2R,4R)-Neoiso-dihydrocarveol | 52.4 | 0.03 |
| MOL000041 | PHA | 41.62 | 0.04 |
| MOL000042 | LPG | 87.69 | 0.01 |
| MOL000043 | atractylenolide i | 37.37 | 0.15 |
| MOL000044 | atractylenolideII | 47.5 | 0.15 |
| MOL000045 | atractylenolide iii | 68.11 | 0.17 |
| MOL000046 | atractylone | 41.1 | 0.13 |
| MOL000047 | juniper camphor | 33.3 | 0.1 |
| MOL000048 | (5E,9Z)-3,6,10-trimethyl-4,7,8,11-tetrahydrocyclodeca[b]furan | 43.17 | 0.1 |
| MOL000049 | 3β-acetoxyatractylone | 54.07 | 0.22 |
| MOL000050 | GLY | 48.74 | 0 |
| MOL000054 | L- | 47.64 | 0.03 |
| MOL000056 | DTY | 57.55 | 0.05 |
| MOL000057 | DIBP | 49.63 | 0.13 |
| MOL000058 | 2-[(2R,5S,6S)-6,10-dimethylspiro[4.5]dec-9-en-2-yl]propan-2-ol | 38.59 | 0.09 |
| MOL000060 | selina-4(14),7(11)-dien-8-one | 32.31 | 0.1 |
| MOL000061 | Prolinum | 77.57 | 0.01 |
| MOL000064 | D-Serin | 83.59 | 0.01 |
| MOL000065 | ASI | 79.74 | 0.02 |
| MOL000066 | alloaromadedrene | 53.46 | 0.1 |
| MOL000067 | L-Valin | 53.33 | 0.01 |
| MOL000068 | L-Ile | 59.05 | 0.02 |
| MOL000070 | Ethyl pivaloylacetate | 40.52 | 0.03 |

| | | | |
|---|---|---|---|
| MOL000071 | Istidina | 53.18 | 0.03 |
| MOL000072 | 8β-ethoxy atractylenolide III | 35.95 | 0.21 |
| MOL000026 | stigmast-22E-en-3beta-ol | 10.39 | 0.75 |
| MOL000062 | biatractylolide | 17.45 | 0.81 |
| MOL000063 | ATRACTYLODES MACROCEPHALA | 14.6 | 0.81 |

**Supplementary Table 3. Chemical compounds of *Saposhnikoviae Radix***

| Mol ID | Chemical Compound | OB (%) | DL |
|---|---|---|---|
| MOL000011 | (2R,3R)-3-(4-hydroxy-3-methoxy-phenyl)-5-methoxy-2-methylol-2,3-dihydropyrano[5,6-h][1,4]benzodioxin-9-one | 68.83 | 0.66 |
| MOL000018 | (+/-)-Isoborneol | 86.98 | 0.05 |
| MOL000023 | Hemo-sol | 39.84 | 0.02 |
| MOL000114 | vanillic acid | 35.47 | 0.04 |
| MOL000115 | Undecenal | 39.35 | 0.03 |
| MOL000116 | Nonanal | 40.28 | 0.02 |
| MOL000118 | (L)-alpha-Terpineol | 48.8 | 0.03 |
| MOL000122 | 1,8-cineole | 39.73 | 0.05 |
| MOL000125 | (-)-alpha-Pinene | 46.25 | 0.05 |
| MOL000131 | EIC | 41.9 | 0.14 |
| MOL000162 | beta-Chamigrene | 31.99 | 0.08 |
| MOL000172 | Furol | 34.35 | 0.01 |
| MOL000173 | wogonin | 30.68 | 0.23 |
| MOL000196 | L-Bornyl acetate | 65.52 | 0.08 |
| MOL000198 | (R)-linalool | 39.8 | 0.02 |
| MOL000202 | Moslene | 33.02 | 0.02 |
| MOL000234 | L-Limonen | 38.09 | 0.02 |
| MOL000266 | beta-Cubebene | 32.81 | 0.11 |
| MOL000268 | (1S,5S)-1-isopropyl-4-methylenebicyclo[3.1.0]hexane | 46.21 | 0.04 |
| MOL000271 | l-carvone | 49.47 | 0.03 |
| MOL000302 | Undekansaeure | 30.14 | 0.03 |
| MOL000358 | beta-sitosterol | 36.91 | 0.75 |
| MOL000359 | sitosterol | 36.91 | 0.75 |
| MOL000474 | (-)-Epoxycaryophyllene | 35.94 | 0.13 |
| MOL000597 | Neryl acetate | 57.47 | 0.04 |
| MOL000614 | osthol | 38.75 | 0.13 |
| MOL000666 | hexanal | 55.71 | 0.01 |
| MOL000668 | PENTYLFURAN | 54.59 | 0.02 |
| MOL000675 | oleic acid | 33.13 | 0.14 |
| MOL000676 | DBP | 64.54 | 0.13 |
| MOL000699 | m-Cymol | 48.85 | 0.02 |
| MOL000708 | WLN: VHR | 32.63 | 0.01 |
| MOL000709 | (S)-Matsutake alcohol | 40.11 | 0.01 |
| MOL000723 | trans-2,4-decadienal | 51.03 | 0.02 |
| MOL000905 | ()-beta-Pinene | 44.77 | 0.05 |
| MOL000914 | (5S)-1-isopropyl-4-methylbicyclo[3.1.0]hex-3-ene | 47.13 | 0.04 |
| MOL000922 | (R)-p-Menth-1-en-4-ol | 32.16 | 0.03 |
| MOL000974 | cuminal | 38.29 | 0.03 |
| MOL001114 | 2-[(1R)-2,2,3-trimethyl-1-cyclopent-3-enyl]ethanal | 45.71 | 0.03 |

| | | | |
|---|---|---|---|
| MOL001121 | 19894-97-4 | 49.98 | 0.06 |
| MOL001129 | l-Verbenone | 50.66 | 0.06 |
| MOL001245 | (1S,5R)-7,7-dimethyl-4-bicyclo[3.1.1]hept-3-enecarboxaldehyde | 41.25 | 0.06 |
| MOL001390 | 49070_FLUKA | 85.51 | 0.12 |
| MOL001494 | Mandenol | 42 | 0.19 |
| MOL001578 | Hypnon | 48.19 | 0.02 |
| MOL001641 | METHYL LINOLEATE | 41.93 | 0.17 |
| MOL001889 | Methyl linolelaidate | 41.93 | 0.17 |
| MOL001940 | Falcarindiol | 39.3 | 0.11 |
| MOL001941 | Ammidin | 34.55 | 0.22 |
| MOL001942 | isoimperatorin | 45.46 | 0.23 |
| MOL001944 | Marmesin | 50.28 | 0.18 |
| MOL001945 | Majudin | 42.21 | 0.13 |
| MOL001949 | panaxynol | 42.44 | 0.1 |
| MOL001950 | psoralen | 33.06 | 0.1 |
| MOL001953 | Uvadex | 35.3 | 0.13 |
| MOL002024 | (1S,4R)-1,7,7-trimethylbicyclo[2.2.1]hept-2-ene | 39.62 | 0.04 |
| MOL002029 | ()-Cuparene | 38.26 | 0.07 |
| MOL002033 | cis-Thujopsene | 56.43 | 0.12 |
| MOL002138 | p-Cymen-8-ol | 32.26 | 0.03 |
| MOL002153 | 1H-Cycloprop(e)azulen-7-ol, decahydro-1,1,7-trimethyl-4-methylene-, (1aR-(1aalpha,4aalpha,7beta,7abeta,7balpha))- | 82.33 | 0.12 |
| MOL002198 | Heptan | 41.8 | 0 |
| MOL002207 | 1(3H)-Isobenzofuranone, 3-butyl-3a,4,5,6-tetrahydro-, cis-(-)- | 65.03 | 0.07 |
| MOL002379 | PTL | 59.53 | 0 |
| MOL002453 | (-)-Comphene | 34.98 | 0.04 |
| MOL002480 | Methylbutenol | 54.58 | 0.01 |
| MOL002520 | .beta.-Fenchyl acetate, exo- | 108.68 | 0.07 |
| MOL002644 | Phellopterin | 40.19 | 0.28 |
| MOL003040 | Amylol | 76.16 | 0 |
| MOL003050 | nonanoic acid | 40.51 | 0.02 |
| MOL003453 | ZINC02571348 | 39.15 | 0.02 |
| MOL003547 | Azaron | 38.39 | 0.06 |
| MOL003588 | Prangenidin | 36.31 | 0.22 |
| MOL003951 | Benzyl isovalerate | 58.44 | 0.05 |
| MOL004480 | acetic acid | 47.87 | 0 |
| MOL004775 | Isobergapten | 34.75 | 0.13 |
| MOL004793 | Marmesine | 84.77 | 0.18 |
| MOL005274 | Neohexane | 37.81 | 0.01 |
| MOL005723 | Phytodolor | 52.32 | 0.1 |
| MOL007502 | NSC692928 | 43.31 | 0.1 |
| MOL007514 | methyl icosa-11,14-dienoate | 39.67 | 0.23 |

| | | | |
|---|---|---|---|
| MOL007545 | lanceol | 37.54 | 0.07 |
| MOL010120 | (1S,4R,4aR,8aR)-1-isopropyl-4,7-dimethyl-2,3,4,5,6,8a-hexahydro-1H-naphthalen-4a-ol | 62.54 | 0.09 |
| MOL010244 | (2S)-Flavanone | 64.04 | 0.13 |
| MOL011387 | heptadeca-1,8-dien-4,6-diyn-3,10-diol | 53.86 | 0.11 |
| MOL011648 | METHYL 10-OCTADECENOATE | 31.9 | 0.17 |
| MOL011730 | 11-hydroxy-sec-o-beta-d-glucosylhamaudol_qt | 50.24 | 0.27 |
| MOL011732 | anomalin | 59.65 | 0.66 |
| MOL011737 | divaricatacid | 87 | 0.32 |
| MOL011738 | divaricataester,a | 64.02 | 0.13 |
| MOL011740 | divaricatol | 31.65 | 0.38 |
| MOL011742 | fraxidin | 42.22 | 0.1 |
| MOL011745 | isofraxidin | 39.75 | 0.1 |
| MOL011746 | isopimpinellin | 43.14 | 0.17 |
| MOL011747 | ledebouriellol | 32.05 | 0.51 |
| MOL011748 | nodakenetin | 68.62 | 0.15 |
| MOL011749 | phelloptorin | 43.39 | 0.28 |
| MOL011753 | 5-O-Methylvisamminol | 37.99 | 0.25 |
| MOL011754 | 4-hydroxy-9-methoxyfuro[3,2-g]chromen-7-one | 31.78 | 0.15 |
| MOL011755 | 5-methoxy-8-hydroxypsoralen | 48.4 | 0.15 |
| MOL011756 | 7-octen-4-ol | 33.99 | 0.01 |
| MOL011860 | d-Dihydrocarvone | 62.94 | 0.03 |
| MOL013077 | Decursin | 39.27 | 0.38 |
| MOL013176 | 2-[(2S)-7-oxo-2,3-dihydrofuro[4,5-g]chromen-2-yl]propan-2-yl (E)-2-methylbut-2-enoate | 17.32 | 0.36 |
| MOL011752 | sec-o-beta-d-glucosylhamaudol | 15.08 | 0.75 |
| MOL011750 | prim-o-beta-d-glucosylcimifugin | 27 | 0.79 |
| MOL011743 | hamaudol | 21.46 | 0.24 |
| MOL011741 | fangfengalpyrimidine | 15.37 | 0.24 |
| MOL011739 | divaricataester,b | 26.1 | 0.39 |
| MOL011736 | 3'-O-angeloylhamaudol | 12.01 | 0.45 |
| MOL011735 | deltoin | 14.25 | 0.36 |
| MOL011734 | Cimifugin | 13.49 | 0.29 |
| MOL011731 | 3'-O-Acetylhamaudol | 26.21 | 0.34 |
| MOL011729 | 11-hydroxy-sec-o-beta-d-glucosylhamaudol | 16.62 | 0.77 |
| MOL009356 | Tectochrysin | 9.57 | 0.2 |
| MOL005786 | Byakangelicin | 27.67 | 0.35 |
| MOL001946 | Ammijin | 15.48 | 0.69 |
| MOL000663 | lignoceric acid | 14.9 | 0.33 |
| MOL000661 | PENTACOSANOIC ACID | 14.57 | 0.37 |
| MOL000373 | (2S)-4-methoxy-7-methyl-2-[1-methyl-1-[(2S,3R,4S,5S,6R)-3,4,5-trihydroxy-6- | 5.38 | 0.81 |

| | | | |
|---|---|---|---|
| | methylol-tetrahydropyran-2-yl]oxy-ethyl]-2,3-dihydrofuro[3,2-g]chromen-5-one | | |
| MOL000357 | Sitogluside | 20.63 | 0.62 |

**Supplementary Table 4. Human target proteins of YPFS**

| NO. | Target Proteins | NO. | Target Proteins | NO. | Target Proteins |
|---|---|---|---|---|---|
| 1 | AARS | 2 | ESR1 | 3 | NCOA2 |
| 4 | ABAT | 5 | ESR2 | 6 | NDUFS1 |
| 7 | ABI1 | 8 | F13A1 | 9 | NDUFS3 |
| 10 | ACADM | 11 | F7 | 12 | NDUFS8 |
| 13 | ACADSB | 14 | FABP1 | 15 | NDUFV1 |
| 16 | ACAT2 | 17 | FASN | 18 | NFS1 |
| 19 | ACE | 20 | FKBP1A | 21 | NLRP3 |
| 22 | ACHE | 23 | FN1 | 24 | NOS1 |
| 25 | ACY1 | 26 | FOLH1 | 27 | NOS2 |
| 28 | ADH1A | 29 | FOS | 30 | NOS3 |
| 31 | ADH1B | 32 | G6PD | 33 | NPL |
| 34 | ADH1C | 35 | GABRA1 | 36 | NQO2 |
| 37 | ADH4 | 38 | GABRA2 | 39 | NR3C1 |
| 40 | ADH7 | 41 | GABRA3 | 42 | NR3C2 |
| 43 | ADRA1A | 44 | GABRA4 | 45 | NTRK2 |
| 46 | ADRA1B | 47 | GABRA5 | 48 | OAT |
| 49 | ADRA1D | 50 | GABRA6 | 51 | OAZ3 |
| 52 | ADRA2A | 53 | GABRB1 | 54 | OPLAH |
| 55 | ADRA2B | 56 | GABRB3 | 57 | OPRM1 |
| 58 | ADRA2C | 59 | GALE | 60 | PAFAH1B3 |
| 61 | ADRB1 | 62 | GALT | 63 | PAM |
| 64 | ADRB2 | 65 | GAMT | 66 | PC |
| 67 | ADSSL1 | 68 | GAP43 | 69 | PCCA |
| 70 | AGXT | 71 | GAPDHS | 72 | PDE3A |
| 73 | AGXT2 | 74 | GATM | 75 | PDHB |
| 76 | AHCY | 77 | GBA2 | 78 | PDX1 |
| 79 | AHSA1 | 80 | GCAT | 81 | PDXP |
| 82 | AKR1B1 | 83 | GCG | 84 | PFKFB1 |
| 85 | AKT1 | 86 | GCLM | 87 | PFKFB4 |
| 88 | ALAD | 89 | GGT5 | 90 | PFKM |
| 91 | ALAS1 | 92 | GIG18 | 93 | PGD |
| 94 | ALAS2 | 95 | GLB1 | 96 | PGLS |
| 97 | ALDH18A1 | 98 | GLDC | 99 | PGR |
| 100 | ALDH1A1 | 101 | GLRA1 | 102 | PHOSPHO1 |
| 103 | ALDH1B1 | 104 | GLRA2 | 105 | PHYKPL |
| 106 | ALDH2 | 107 | GLRA3 | 108 | PIK3CG |
| 109 | ALDH4A1 | 110 | GLUD1 | 111 | PIM1 |
| 112 | ALDH5A1 | 113 | GLUD2 | 114 | PIPOX |
| 115 | ALDH9A1 | 116 | GLUL | 117 | PKIA |
| 118 | ALDOA | 119 | GLYAT | 120 | PLA2G1B |
| 121 | ALOX5 | 122 | GLYATL1 | 123 | PLA2G2A |
| 124 | AMD1 | 125 | GM2A | 126 | PLA2G2E |
| 127 | AMT | 128 | GNMT | 129 | PLAU |
| 130 | AMY2A | 131 | GNPDA1 | 132 | PLG |
| 133 | ANXA3 | 134 | GNRH1 | 135 | PNMT |

| | | | | | |
|---|---|---|---|---|---|
| 136 | APRT | 137 | GOT2 | 138 | PNP |
| 139 | AR | 140 | GPHN | 141 | PON1 |
| 142 | ARF1 | 143 | GPI | 144 | PPARA |
| 145 | ARF4 | 146 | GPT | 147 | PPARD |
| 148 | ARG1 | 149 | GPT2 | 150 | PPARG |
| 151 | ARG2 | 152 | GRIA2 | 153 | PPIA |
| 154 | ASL | 155 | GRIN1 | 156 | PPIH |
| 157 | ASNA1 | 158 | GRIN2A | 159 | PPOX |
| 160 | ASRGL1 | 161 | GRIN2B | 162 | PRDX2 |
| 163 | ATP5A1 | 164 | GRIN2C | 165 | PRKACA |
| 166 | BAAT | 167 | GSK3B | 168 | PRKCD |
| 169 | BAX | 170 | GSR | 171 | PROSC |
| 172 | BBC3 | 173 | GSTA1 | 174 | PRSS1 |
| 175 | BCHE | 176 | GSTA2 | 177 | PRSS3 |
| 178 | BCL2 | 179 | GSTA5 | 180 | PSMA1 |
| 181 | BDNF | 182 | GSTM1 | 183 | PTGER3 |
| 184 | BHMT | 185 | GSTM4 | 186 | PTGS1 |
| 187 | BHMT2 | 188 | GSTP1 | 189 | PTGS2 |
| 190 | C8G | 191 | GSTZ1 | 192 | PTPN1 |
| 193 | CA2 | 194 | GUSB | 195 | PYCR1 |
| 196 | CA4 | 197 | HAGH | 198 | PYCR2 |
| 199 | CAD | 200 | HAO1 | 201 | PYGL |
| 202 | CALM1 | 203 | HARS | 204 | PYGM |
| 205 | CASP3 | 206 | HDAC8 | 207 | PYY |
| 208 | CASP8 | 209 | HDC | 210 | RB1 |
| 211 | CASP9 | 212 | HMGCR | 213 | RBKS |
| 214 | CAT | 215 | HMOX1 | 216 | RBP2 |
| 217 | CBR1 | 218 | HS3ST3A1 | 219 | RDH11 |
| 220 | CBS | 221 | HSD17B1 | 222 | RDH13 |
| 223 | CCK | 224 | HSD17B8 | 225 | RDH5 |
| 226 | CCL2 | 227 | HTR2A | 228 | RELA |
| 229 | CCNA2 | 230 | HTR3A | 231 | REN |
| 232 | CCND1 | 233 | IGHG1 | 234 | RNASE1 |
| 235 | CD36 | 236 | IKBKB | 237 | RRM1 |
| 238 | CDC25B | 239 | IL10 | 240 | RTCA |
| 241 | CDK2 | 242 | IL1B | 243 | RXRA |
| 244 | CDK4 | 245 | IL6 | 246 | RXRG |
| 247 | CDKN1A | 248 | IMPA1 | 249 | SCD |
| 250 | CELA1 | 251 | INS | 252 | SCN5A |
| 253 | CES2 | 254 | ISYNA1 | 255 | SDHA |
| 256 | CETP | 257 | JUN | 258 | SDS |
| 259 | CHDH | 260 | KCNH2 | 261 | SELP |
| 262 | CHEK1 | 263 | KCNMA1 | 264 | SERPINA1 |
| 265 | CHRM1 | 266 | KDR | 267 | SERPINE1 |
| 268 | CHRM2 | 269 | KYAT1 | 270 | SHMT1 |
| 271 | CHRM3 | 272 | KYNU | 273 | SHMT2 |

| | | | | | |
|---|---|---|---|---|---|
| 274 | CHRM4 | 275 | LARS | 276 | SI |
| 277 | CHRNA2 | 278 | LCMT2 | 279 | SIRT5 |
| 280 | CHRNA7 | 281 | LCT | 282 | SLC25A10 |
| 283 | CITED1 | 284 | LDHA | 285 | SLC25A12 |
| 286 | CKM | 287 | LDHB | 288 | SLC25A13 |
| 289 | COX5A | 290 | LPL | 291 | SLC25A15 |
| 292 | CPA1 | 293 | LTA4H | 294 | SLC2A2 |
| 295 | CPB1 | 296 | MAOA | 297 | SLC36A1 |
| 298 | CPT1A | 299 | MAOB | 300 | SLC6A2 |
| 301 | CRH | 302 | MAP1B | 303 | SLC6A3 |
| 304 | CRP | 305 | MAP2 | 306 | SLC6A4 |
| 307 | CTH | 308 | MAPK10 | 309 | SOAT1 |
| 310 | CTNNB1 | 311 | MAPK14 | 312 | SOD1 |
| 313 | CTRB1 | 314 | MARS | 315 | SPR |
| 316 | CTSB | 317 | MAT1A | 318 | SRC |
| 319 | CTSD | 320 | MAT2A | 321 | SULT2B1 |
| 322 | CXCL8 | 323 | MCAT | 324 | TEP1 |
| 325 | DAO | 326 | MCL1 | 327 | TGFB1 |
| 328 | DCXR | 329 | ME1 | 330 | TGM7 |
| 331 | DDAH2 | 332 | ME2 | 333 | TNF |
| 334 | DHODH | 335 | ME3 | 336 | TNFSF13B |
| 337 | DHRSX | 338 | METAP2 | 339 | TP53 |
| 340 | DMPK | 341 | MGAM | 342 | TPI1 |
| 343 | DNPEP | 344 | MGMT | 345 | TRAPPC4 |
| 346 | DPP4 | 347 | MMP1 | 348 | TREH |
| 349 | DRD1 | 350 | MMP12 | 351 | TRPV1 |
| 352 | DUOX2 | 353 | MMP3 | 354 | TXNRD1 |
| 355 | EDN1 | 356 | MMP8 | 357 | TYRP1 |
| 358 | EGLN1 | 359 | MPO | 360 | UCP2 |
| 361 | EIF6 | 362 | MTR | 363 | UCP3 |
| 364 | ENPEP | 365 | NAGS | 366 | UROD |
| 367 | EPHX1 | 368 | NCF1 | 369 | WARS2 |
| 370 | ERBB2 | 371 | NCOA1 | 372 | XDH |

**Supplementary Table 5. Asthma-related human genes**

| NO. | Genes | NO. | Genes | NO. | Genes | NO. | Genes |
|---|---|---|---|---|---|---|---|
| 1 | IL13 | 2 | HNMT | 3 | JAK1 | 4 | ITK |
| 5 | NPSR1 | 6 | T | 7 | ATP2A2 | 8 | TNFRSF14 |
| 9 | IL33 | 10 | CRB1 | 11 | CYSLTR1 | 12 | CRHR2 |
| 13 | TSLP | 14 | P2RX7 | 15 | STAT5A | 16 | RNASE2 |
| 17 | HLA-DQB1 | 18 | HAVCR1 | 19 | IFNA1 | 20 | PTGER3 |
| 21 | TNF | 22 | IL15 | 23 | ITGA4 | 24 | GRK5 |
| 25 | IL1RL1 | 26 | IL12A | 27 | CRHR1 | 28 | LTB4R |
| 29 | RAD50 | 30 | VCAM1 | 31 | TBXA2R | 32 | MIR15A |
| 33 | CHI3L1 | 34 | CD274 | 35 | PROS1 | 36 | PMCH |
| 37 | SMAD3 | 38 | CYP1B1 | 39 | MYB | 40 | SLC25A46 |
| 41 | ORMDL3 | 42 | AHR | 43 | NR3C2 | 44 | DENND1B |
| 45 | IL18R1 | 46 | ASOBS | 47 | IL7 | 48 | TLR10 |
| 49 | ADRB2 | 50 | ASRT8 | 51 | EMSY | 52 | BMPR1B |
| 53 | GSDMB | 54 | ASRT6 | 55 | GSTA1 | 56 | ABI3BP |
| 57 | HLA-G | 58 | ASRT4 | 59 | IL27 | 60 | CCL3L1 |
| 61 | VEGFA | 62 | ASRT3 | 63 | STARD13 | 64 | GSTO2 |
| 65 | TGFB1 | 66 | AASTH27 | 67 | ALOX5AP | 68 | SVEP1 |
| 69 | HLA-DRA | 70 | AASTH54 | 71 | BMPR2 | 72 | SERPINE2 |
| 73 | ACE | 74 | AASTH55 | 75 | PEBP1 | 76 | TAGLN |
| 77 | FLG | 78 | AASTH38 | 79 | IL9 | 80 | LTBP1 |
| 81 | IL10 | 82 | AASTH17 | 83 | SCGB3A2 | 84 | IL31 |
| 85 | IL6 | 86 | AASTH31 | 87 | SLC11A1 | 88 | CCR4 |
| 89 | ADIPOQ | 90 | AASTH7 | 91 | ADCY3 | 92 | IL20 |
| 93 | MMP9 | 94 | AASTH42 | 95 | TAF7 | 96 | NEIL1 |
| 97 | HLA-DRB1 | 98 | AASTH41 | 99 | PLA2G2A | 100 | HAS2 |
| 101 | BDNF | 102 | AASTH15 | 103 | KITLG | 104 | PCDH9 |
| 105 | VDR | 106 | AASTH16 | 107 | HAVCR2 | 108 | CCL7 |
| 109 | HLA-DQA1 | 110 | AASTH39 | 111 | DEFB1 | 112 | CRLF2 |
| 113 | TLR4 | 114 | AASTH2 | 115 | CTTN | 116 | SPATS2L |
| 117 | RORA | 118 | AASTH3 | 119 | IKZF2 | 120 | IL19 |
| 121 | IL1B | 122 | AASTH4 | 123 | SFTPA1 | 124 | GLRX |
| 125 | ALOX5 | 126 | AASTH5 | 127 | NAT1 | 128 | MIR19A |
| 129 | GSTT1 | 130 | AASTH6 | 131 | STAT4 | 132 | FBXL7 |
| 133 | CRP | 134 | AASTH1 | 135 | CHRM3 | 136 | PCDH20 |
| 137 | GSTM1 | 138 | AASTH28 | 139 | MMP12 | 140 | FLT3LG |
| 141 | IFNG | 142 | AASTH32 | 143 | ANXA5 | 144 | MKLN1 |
| 145 | MTHFR | 146 | AASTH56 | 147 | ADAM12 | 148 | PTPRD |
| 149 | IL6R | 150 | AASTH29 | 151 | USP15 | 152 | IL17RA |
| 153 | HLA-DPB1 | 154 | AASTH37 | 155 | PLA2G4A | 156 | KCNMB1 |
| 157 | CXCL8 | 158 | AASTH33 | 159 | IL6ST | 160 | TFF2 |
| 161 | APOE | 162 | AASTH36 | 163 | CCL3 | 164 | FABP5 |
| 165 | EGFR | 166 | AASTH35 | 167 | RYR2 | 168 | EFNA1 |
| 169 | SERPINE1 | 170 | AASTH34 | 171 | ITGB4 | 172 | BPI |
| 173 | PTGS2 | 174 | AASTH10 | 175 | CD86 | 176 | MYH11 |

| | | | | | | | |
|---|---|---|---|---|---|---|---|
| 177 | CFTR | 178 | AASTH8 | 179 | SYNE2 | 180 | CNTLN |
| 181 | IL2RA | 182 | AASTH11 | 183 | EDNRB | 184 | TET1 |
| 185 | GSTP1 | 186 | AASTH13 | 187 | MPL | 188 | TAC3 |
| 189 | CDK2 | 190 | AASTH12 | 191 | RACK1 | 192 | IKZF4 |
| 193 | LEP | 194 | AASTH9 | 195 | AREG | 196 | C10orf54 |
| 197 | IL5 | 198 | AASTH22 | 199 | TNFSF4 | 200 | LGALS7 |
| 201 | STAT3 | 202 | AASTH24 | 203 | CSF3 | 204 | PDCD1LG2 |
| 205 | TBX21 | 206 | AASTH18 | 207 | P2RY12 | 208 | AOC1 |
| 209 | CCL2 | 210 | AASTH19 | 211 | NOD1 | 212 | ADH5 |
| 213 | SLC6A4 | 214 | AASTH23 | 215 | TUSC3 | 216 | LTB4R2 |
| 217 | NR3C1 | 218 | AASTH26 | 219 | PDCD4 | 220 | GZMA |
| 221 | IL17A | 222 | AASTH25 | 223 | CSF1R | 224 | CLCA1 |
| 225 | GC | 226 | AASTH21 | 227 | DGKH | 228 | DOCK10 |
| 229 | IL18 | 230 | AASTH20 | 231 | TERC | 232 | CDHR3 |
| 233 | S100B | 234 | AASTH46 | 235 | CFL1 | 236 | HLX |
| 237 | NOS3 | 238 | AASTH45 | 239 | TJP1 | 240 | XCL1 |
| 241 | CCL11 | 242 | AASTH44 | 243 | C5 | 244 | PLA2G10 |
| 245 | IL4 | 246 | AASTH53 | 247 | VTN | 248 | ICOSLG |
| 249 | CCR5 | 250 | AASTH43 | 251 | CCL20 | 252 | SLURP1 |
| 253 | BTNL2 | 254 | AASTH51 | 255 | CHRNA5 | 256 | C3AR1 |
| 257 | ICAM1 | 258 | AASTH50 | 259 | SLC24A2 | 260 | IL13RA1 |
| 261 | TNXB | 262 | AASTH49 | 263 | MS4A2 | 264 | SERPINB4 |
| 265 | PPARG | 266 | AASTH47 | 267 | RAB11FIP2 | 268 | ZBTB10 |
| 269 | IRF1 | 270 | AASTH48 | 271 | CCL18 | 272 | TRPV2 |
| 273 | GSDMA | 274 | AASTH52 | 275 | DUSP1 | 276 | EBI3 |
| 277 | MYCN | 278 | AASTH14 | 279 | MCAM | 280 | HHIP |
| 281 | CTLA4 | 282 | AASTH40 | 283 | CHRNA3 | 284 | DLEU1 |
| 285 | IL1R1 | 286 | AASTH30 | 287 | MYLK | 288 | MAP2K3 |
| 289 | TLR2 | 290 | CASR | 291 | TLR5 | 292 | RAP1GAP2 |
| 293 | CDH1 | 294 | F2RL1 | 295 | HSP90B1 | 296 | PDE11A |
| 297 | HIF1A | 298 | MIR155 | 299 | IL3 | 300 | TIMD4 |
| 301 | PTEN | 302 | IL5RA | 303 | ENPP2 | 304 | KLHL5 |
| 305 | FOXP3 | 306 | ANGPT2 | 307 | TLR8 | 308 | IL9R |
| 309 | NFKB1 | 310 | SCGB1A1 | 311 | TREM1 | 312 | CCL15 |
| 313 | CD14 | 314 | CREB1 | 315 | CCR3 | 316 | CCL1 |
| 317 | ABCB1 | 318 | ANK1 | 319 | FTL | 320 | CCL24 |
| 321 | PLA2G7 | 322 | TGM2 | 323 | TLR6 | 324 | BST1 |
| 325 | SOX9 | 326 | ACO1 | 327 | INHA | 328 | ZNF71 |
| 329 | CTNNB1 | 330 | ADAM33 | 331 | KANK1 | 332 | NTF4 |
| 333 | PDE4D | 334 | IDO1 | 335 | CXCR1 | 336 | TRG |
| 337 | ERBB4 | 338 | SELE | 339 | TSPO | 340 | EFR3B |
| 341 | MBL2 | 342 | PROC | 343 | ITLN1 | 344 | IGSF3 |
| 345 | COMT | 346 | KLK3 | 347 | ACKR1 | 348 | RGS5 |
| 349 | PRKCE | 350 | MIR146A | 351 | FCER2 | 352 | COMMD10 |
| 353 | HLA-E | 354 | NOS1 | 355 | CCL17 | 356 | RAMP1 |
| 357 | CCL5 | 358 | CTNNA3 | 359 | S1PR1 | 360 | PYHIN1 |
| 361 | CD4 | 362 | CCR2 | 363 | CMA1 | 364 | KIF3A |

| | | | | | | | |
|---|---|---|---|---|---|---|---|
| 365 | PON1 | 366 | CALCA | 367 | C4A | 368 | NAGA |
| 369 | PTHLH | 370 | XIAP | 371 | ICOS | 372 | MIR192 |
| 373 | SPP1 | 374 | ADORA2A | 375 | IL16 | 376 | DPP10 |
| 377 | BCL2 | 378 | IRAK3 | 379 | CHRM2 | 380 | ADAMTS9 |
| 381 | HMGB1 | 382 | FCGR2A | 383 | HERC5 | 384 | DAP3 |
| 385 | ITGB3 | 386 | RNASE3 | 387 | HTR3A | 388 | SEMA7A |
| 389 | MMP2 | 390 | CAMP | 391 | SYNPO2 | 392 | DLEU7 |
| 393 | WDR36 | 394 | AGL | 395 | SLPI | 396 | GLCCI1 |
| 397 | NFE2L2 | 398 | POSTN | 399 | IFNL1 | 400 | CD53 |
| 401 | CXCR4 | 402 | NFKBIA | 403 | C5AR1 | 404 | DEUP1 |
| 405 | HMOX1 | 406 | TGFBR1 | 407 | PTGER4 | 408 | SLC24A3 |
| 409 | IL1RN | 410 | SLC26A4 | 411 | CYP11A1 | 412 | MINA |
| 413 | SOD1 | 414 | ITGB2 | 415 | LPIN2 | 416 | MMP28 |
| 417 | IL12B | 418 | TGFBR2 | 419 | TRPM8 | 420 | SSH1 |
| 421 | MAPK1 | 422 | IKZF3 | 423 | IRF4 | 424 | LEPQTL1 |
| 425 | CYP2C19 | 426 | S100A9 | 427 | PBX2 | 428 | IL18RAP |
| 429 | SOD2 | 430 | FCGR3A | 431 | RGS2 | 432 | FAM19A2 |
| 433 | CCND1 | 434 | ANXA2 | 435 | KIR2DS2 | 436 | SERBP1 |
| 437 | TLR1 | 438 | G6PD | 439 | CRIM1 | 440 | SPTBN2 |
| 441 | PTGDR | 442 | FGB | 443 | PTGDR2 | 444 | ACSL3 |
| 445 | CDH13 | 446 | CYBA | 447 | XDH | 448 | HPGDS |
| 449 | CXCL12 | 450 | TNFSF13B | 451 | AKR1C3 | 452 | PTPRE |
| 453 | APOA1 | 454 | CDH23 | 455 | TNS1 | 456 | ZNF365 |
| 457 | GATA2 | 458 | ADM | 459 | OSM | 460 | IL17RB |
| 461 | PRKCQ | 462 | FYN | 463 | PDE9A | 464 | NFIA |
| 465 | MMP1 | 466 | MUSK | 467 | CHRM1 | 468 | SIGLEC8 |
| 469 | PARP1 | 470 | MMP7 | 471 | LAMC2 | 472 | MMP25 |
| 473 | SLC30A8 | 474 | SH2B3 | 475 | DENND1A | 476 | XKR6 |
| 477 | EDN1 | 478 | EGR1 | 479 | ATG3 | 480 | ENPP3 |
| 481 | SLC22A5 | 482 | F13A1 | 483 | KIR2DL2 | 484 | RETNLB |
| 485 | XRCC1 | 486 | LGALS1 | 487 | TPSAB1 | 488 | ACAA1 |
| 489 | KIT | 490 | PTX3 | 491 | FADS2 | 492 | SIGIRR |
| 493 | AGER | 494 | CSMD1 | 495 | HRH1 | 496 | CCL28 |
| 497 | CXCL10 | 498 | SELP | 499 | TRPA1 | 500 | CPA3 |
| 501 | IL4R | 502 | NPY | 503 | SCG3 | 504 | KIFC1 |
| 505 | CAV1 | 506 | FGA | 507 | EDIL3 | 508 | RANBP6 |
| 509 | CD44 | 510 | RBP4 | 511 | PLD2 | 512 | NPS |
| 513 | MIR21 | 514 | F2R | 515 | RGS4 | 516 | SLC26A9 |
| 517 | ITGB1 | 518 | EPHX1 | 519 | HEXB | 520 | MMP21 |
| 521 | NOTCH4 | 522 | NPSR1-AS1 | 523 | SFTPC | 524 | SOCS5 |
| 525 | MAVS | 526 | SOCS3 | 527 | CD276 | 528 | MUC19 |
| 529 | NAT2 | 530 | IL23R | 531 | FPR2 | 532 | PCDH1 |
| 533 | PLAUR | 534 | ADRB3 | 535 | IGHE | 536 | TNFAIP8L2 |
| 537 | TLR9 | 538 | VAV3 | 539 | C6orf10 | 540 | UNC119 |
| 541 | LEPR | 542 | TXN | 543 | SPINK5 | 544 | UBE3C |
| 545 | GHRL | 546 | DCLK1 | 547 | RBM17 | 548 | C5orf56 |

| | | | | | | | |
|---|---|---|---|---|---|---|---|
| 549 | NOS2 | 550 | LRP1 | 551 | LTA4H | 552 | SLC30A4 |
| 553 | KDR | 554 | PARK7 | 555 | LGALS9 | 556 | SEMA4A |
| 557 | NOD2 | 558 | CEBPB | 559 | SPRY2 | 560 | DCBLD2 |
| 561 | BAX | 562 | LTF | 563 | GNGT1 | 564 | LYNX1 |
| 565 | CASP3 | 566 | CLEC16A | 567 | CCR1 | 568 | PPP1R12B |
| 569 | CYP1A1 | 570 | SFTPD | 571 | TLE4 | 572 | PSORS1C1 |
| 573 | PTGER2 | 574 | IL22 | 575 | LY96 | 576 | KCNIP4 |
| 577 | PSAP | 578 | FGG | 579 | TGFBR3 | 580 | BATF |
| 581 | LRRC32 | 582 | IL21 | 583 | IL25 | 584 | C1orf100 |
| 585 | PRKG1 | 586 | HSPD1 | 587 | MIR16-1 | 588 | FOXA3 |
| 589 | MAPK3 | 590 | NGF | 591 | PAFAH1B1 | 592 | LY86 |
| 593 | IL2 | 594 | HLA-DQA2 | 595 | CYP2J2 | 596 | INPP4A |
| 597 | HLA-DPA1 | 598 | SMAD2 | 599 | UTS2 | 600 | TPSB2 |
| 601 | FMR1 | 602 | ANGPT1 | 603 | STAT2 | 604 | CHML |
| 605 | F3 | 606 | HDAC2 | 607 | ZBTB38 | 608 | TBCD |
| 609 | SLC22A4 | 610 | NPPA | 611 | ADAM8 | 612 | C6orf118 |
| 613 | HTR2A | 614 | DDX1 | 615 | CCL22 | 616 | CA10 |
| 617 | PRKAA2 | 618 | ADH1B | 619 | ACVR1 | 620 | C11orf71 |
| 621 | P2RY2 | 622 | HLA-DOA | 623 | SDC4 | 624 | TAC4 |
| 625 | TNFSF10 | 626 | MYD88 | 627 | BDKRB1 | 628 | CRTAM |
| 629 | STAT6 | 630 | CTNND2 | 631 | CTNNA1 | 632 | STK10 |
| 633 | CAT | 634 | CX3CR1 | 635 | ADORA1 | 636 | PGAP3 |
| 637 | CFH | 638 | CD28 | 639 | ATF6B | 640 | OR6X1 |
| 641 | CHD7 | 642 | F10 | 643 | KIR2DL3 | 644 | COL6A5 |
| 645 | LTA | 646 | ETS1 | 647 | TNFRSF13B | 648 | ZNF665 |
| 649 | C3 | 650 | MUC5AC | 651 | GCLC | 652 | LOC727896 |
| 653 | FGF2 | 654 | LTC4S | 655 | KIR2DL4 | 656 | DTD1 |
| 657 | CYP3A4 | 658 | SOCS1 | 659 | NDFIP1 | 660 | OPN3 |
| 661 | IL2RB | 662 | CD38 | 663 | PDHA1 | 664 | TPSD1 |
| 665 | PRKCA | 666 | PTPN6 | 667 | MAP3K1 | 668 | FGFBP2 |
| 669 | NQO1 | 670 | TLR7 | 671 | HTR4 | 672 | SEMA3D |
| 673 | CASP8 | 674 | IL17F | 675 | XPR1 | 676 | LOC101060400 |
| 677 | IL1A | 678 | PCDH15 | 679 | SERPINB3 | 680 | CRCT1 |
| 681 | LGALS3 | 682 | ADA | 683 | RIPK2 | 684 | COL26A1 |
| 685 | GAB1 | 686 | CXCR3 | 687 | MRC1 | 688 | RASGRP4 |
| 689 | RETN | 690 | CHRNA7 | 691 | IL37 | 692 | ZPBP2 |
| 693 | MPO | 694 | SIM2 | 695 | TPT1 | 696 | KLK9 |
| 697 | ABCA1 | 698 | MUC7 | 699 | UGT1A6 | 700 | GNG5P5 |
| 701 | TLN1 | 702 | S100A8 | 703 | CD27 | 704 | HCG23 |
| 705 | RHOA | 706 | PECAM1 | 707 | CCL26 | 708 | RPS28P1 |
| 709 | COL1A1 | 710 | ARG1 | 711 | FCRL3 | 712 | LOC101928284 |
| 713 | ABL1 | 714 | APOH | 715 | IRAK4 | 716 | ATPAF1 |
| 717 | MIF | 718 | COL1A2 | 719 | LAMA3 | 720 | IGES |
| 721 | PLAU | 722 | GZMB | 723 | INSIG2 | 724 | HLA-DRB2 |
| 725 | SERPINA1 | 726 | ALOX15 | 727 | TNFRSF4 | 728 | FSIP1 |
| 729 | IGFBP3 | 730 | PTGS1 | 731 | CASP10 | 732 | RGS7BP |
| 733 | EGF | 734 | NKX2-1 | 735 | ATG5 | 736 | ASB3 |

| | | | | | | | |
|---|---|---|---|---|---|---|---|
| 737 | OPRK1 | 738 | BST2 | 739 | ARG2 | 740 | PKDCC |
| 741 | NLRP3 | 742 | TNC | 743 | CLDN5 | 744 | LOC101929231 |
| 745 | FN1 | 746 | TAC1 | 747 | HRH4 | 748 | LOC101928940 |
| 749 | CD40 | 750 | PHF11 | 751 | CD69 | 752 | SPRR2B |
| 753 | TIMP1 | 754 | ELN | 755 | PLXNA4 | 756 | CNKSR3 |
| 757 | FASLG | 758 | CHIT1 | 759 | LAMB3 | 760 | LOC102724802 |
| 761 | CYP3A5 | 762 | ELANE | 763 | IL1R2 | 764 | TPSG1 |
| 765 | CTGF | 766 | INHBA | 767 | NTF3 | 768 | MRPL42 |
| 769 | PMEL | 770 | TNFRSF10B | 771 | SLC6A11 | 772 | SLC6A7 |
| 773 | BCL2L1 | 774 | NOX4 | 775 | FBLN1 | 776 | SCGB1C1 |
| 777 | GATA3 | 778 | TGFB2 | 779 | ADCYAP1R1 | 780 | CACNG6 |
| 781 | CLU | 782 | HPSE2 | 783 | CYSLTR2 | 784 | ZNF432 |
| 785 | CPS1 | 786 | CXCR2 | 787 | FLRT2 | 788 | LYRM9 |
| 789 | TRPV1 | 790 | FCER1A | 791 | CHIA | 792 | MIR3162 |
| 793 | NEWENTRY | | | | | | |

## 1.2 Supplementary Figures

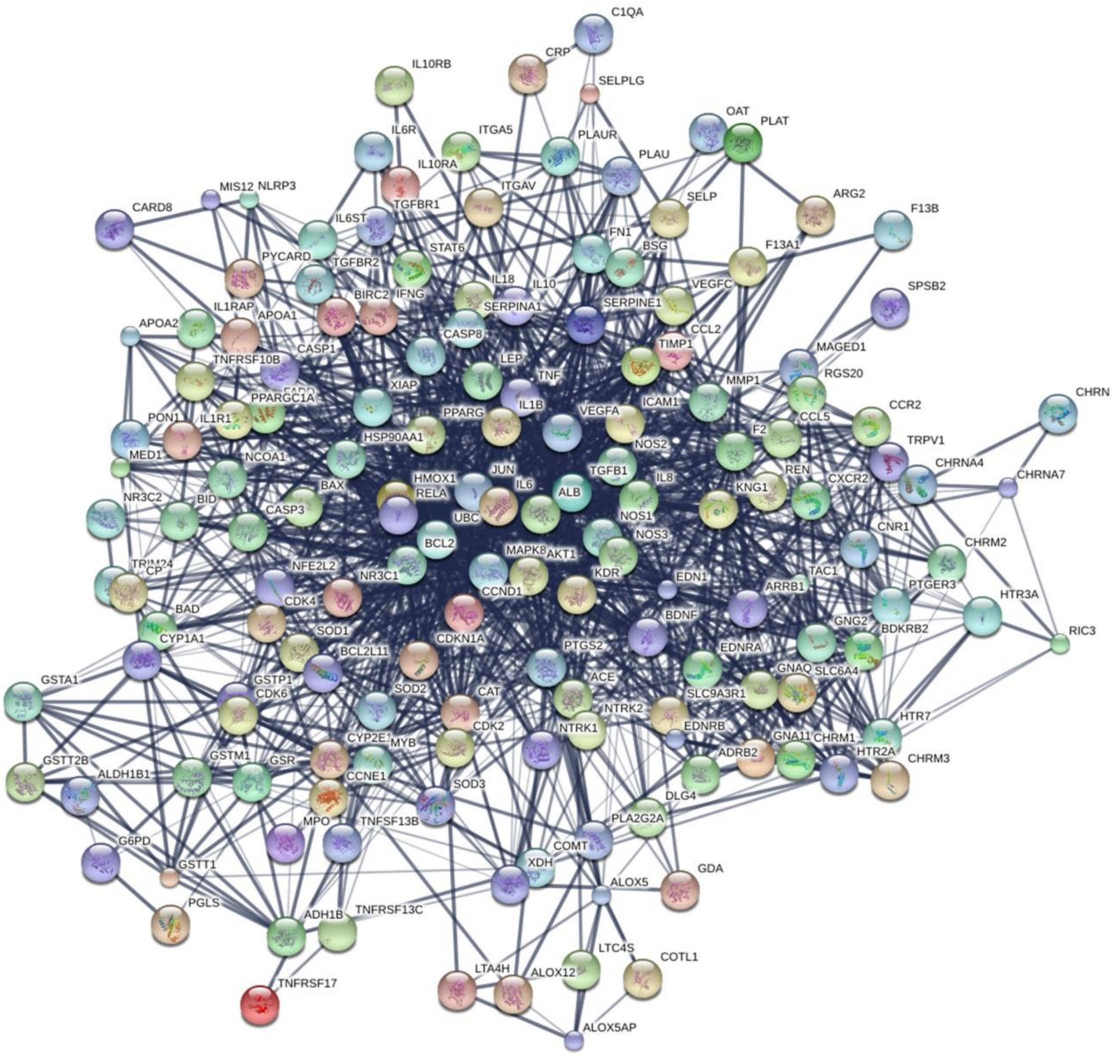


**Supplementary Figure 1. Reaction network of YPFS that antagonizing asthma.** The reaction network of YPFS antagonizing asthma was built through String database. This network contains 158 nodes and 1730 interactive relationships. The thickness of the lines has positive correlation with interactive relationships between YPFS and asthma.
